# Evaluation of Type 2 Diabetes Risk in Individuals With or Without Metabolically Healthy Obesity

**DOI:** 10.3390/biology14060608

**Published:** 2025-05-26

**Authors:** Miguel García Samuelsson, Pedro Juan Tárraga López, Ángel Arturo López-González, Carla Busquets-Cortés, Joan Obrador de Hevia, José Ignacio Ramírez-Manent

**Affiliations:** 1 ADEMA-Health Group, University Institute of Health Sciences (IUNICS), 07120 Palma de Mallorca, Spain; miguelsamuelsson@gmail.com (M.G.S.); c.busquets@eua.edu.es (C.B.-C.); j.obrador@eua.edu.es (J.O.d.H.); jignacioramirez@telefonica.net (J.I.R.-M.); 2 Faculty of Dentistry, ADEMA-UIB University School, Balearic Islands, 07009 Palma de Mallorca, Spain; 3 Faculty of Medicine, University of Castilla-La Mancha, 02071 Albacete, Spain; pjtarraga@sescam.jccm.es; 4 Balearic Islands Health Research Institute Foundation (IDISBA), 07004 Palma de Mallorca, Spain; 5 Balearic Islands Health Service, 07003 Palma de Mallorca, Spain; 6 Faculty of Medicine, University of the Balearic Islands, 07122 Palma de Mallorca, Spain

**Keywords:** metabolically healthy obesity, type 2 diabetes risk, metabolic syndrome, obesity phenotypes, population-based study, diabetes risk scales

## Abstract

Background: The concept of metabolically healthy obesity (MHO) has emerged as a controversial yet clinically relevant phenotype, challenging the traditional view that all obese individuals are uniformly at high metabolic risk. However, the long-term health implications of MHO, particularly its association with type 2 diabetes mellitus (T2DM), remain poorly defined. Objective: To assess the risk of T2DM in individuals with MHO compared to those with metabolically non-healthy obesity (MNHO), using multiple validated diabetes risk scales across a large population-based cohort. Methods: This cross-sectional study analyzed data from over 68,884 adults stratified by sex and BMI-defined obesity status. Metabolic health was categorized using three progressively inclusive definitions based on the number of metabolic syndrome components. Diabetes risk was estimated using Finrisk, Canrisk, TRAQ-D, Thai, Oman, and QD-score tools. Multinomial logistic regression assessed associations between MHO/MNHO phenotypes and high-risk diabetes scores, adjusting for demographic, socioeconomic, and lifestyle variables. Results: Across all definitions and scales, MNHO individuals exhibited significantly higher mean diabetes risk scores and greater prevalence of high-risk categories compared to MHO participants (*p* < 0.001). Nevertheless, MHO individuals consistently showed elevated diabetes risk relative to non-obese, metabolically healthy controls. Physical inactivity, low educational level, lower socioeconomic status, smoking, and absence of a Mediterranean diet were independently associated with higher diabetes risk. MNHO phenotype was a strong and consistent predictor of high-risk classification across all models. Conclusions: While MHO individuals present a comparatively lower risk of T2DM than their MNHO counterparts, their risk remains substantially higher than that of non-obese individuals. These findings highlight the need to refine risk stratification approaches beyond BMI alone, and to consider metabolic health status in guiding prevention strategies for T2DM.

## Simple Summary

Not all people with obesity are equally at risk for type 2 diabetes. Some individuals, although obese, do not show the typical signs of poor metabolic health—like high blood pressure, high blood sugar, or abnormal cholesterol levels. These individuals are known as “metabolically healthy obese” (MHO). This study looked at over 68,000 Spanish workers and compared MHO individuals to those with unhealthy obesity (MNHO). Researchers used several tools to estimate the risk of developing type 2 diabetes. They found that while MHO people had a lower risk than MNHO ones, their risk was still higher than people of normal weight with good metabolic health. The study also showed that lack of physical activity, poor diet, smoking, and low education levels were linked to higher diabetes risk. In short, even if someone with obesity appears healthy now, they may still face serious health risks later. The study highlights the importance of healthy habits and shows that weight alone is not enough to determine a person’s risk for diabetes.

## 1. Introduction

Obesity has emerged as one of the most pressing public health challenges of the 21st century, reaching global epidemic proportions and placing a significant burden on healthcare systems worldwide. According to the World Health Organization (WHO), the global prevalence of obesity has tripled since 1975, with over 650 million adults affected in 2016 alone [1]. This upward trend continues unabated, raising serious concern due to the strong and well-documented links between obesity and a wide array of non-communicable diseases (NCDs), including type 2 diabetes (T2DM), cardiovascular diseases (CVD), certain cancers, musculoskeletal disorders, and mental health disturbances [2,3]. The detrimental health consequences of obesity are multifactorial, involving complex interactions among genetic predispositions, environmental influences, behavioral factors, and metabolic dysfunctions [4].

Despite the robust body of evidence associating excess adiposity with adverse health outcomes, recent years have witnessed the emergence of a seemingly paradoxical phenotype—referred to as metabolically healthy obesity (MHO)—which challenges traditional assumptions regarding the invariable association between obesity and metabolic risk [5,6]. Obesity is typically defined by an excessive accumulation of adipose tissue, most commonly assessed via body mass index (BMI), with a threshold of ≥30 kg/m^2^ indicating clinical obesity [7]. However, BMI alone fails to capture crucial dimensions of metabolic health, such as fat distribution, adipose tissue function, and underlying metabolic alterations [8]. Conventionally, greater adiposity has been presumed to entail higher metabolic dysfunction risk. Nevertheless, a subset of individuals with elevated BMI exhibits a relatively favorable metabolic profile. These so-called MHO individuals show no evidence of the hallmark metabolic abnormalities commonly linked with obesity, such as insulin resistance, dyslipidemia, hypertension, and systemic inflammation [9,10,11].

This phenotype has sparked considerable debate within the scientific and medical communities, particularly regarding its clinical relevance, underlying mechanisms, and long-term prognostic implications [12]. Identifying MHO individuals carries important consequences for risk stratification, therapeutic decision-making, and public health strategy development. If certain people with obesity are not at equivalent metabolic risk as their metabolically unhealthy counterparts (MUO), the prevailing one-size-fits-all approach to obesity management may require reevaluation in favor of a more nuanced understanding of adiposity and its varied health outcomes [13].

Nonetheless, the existence and temporal stability of the MHO phenotype remain subjects of ongoing controversy. Longitudinal investigations have shown that MHO status may be transient, with many individuals transitioning to a metabolically unhealthy state over time [14,15].

Recent data indicate that, even in the absence of overt metabolic alterations, individuals with metabolically healthy obesity are at increased risk of subclinical atherosclerosis, left ventricular dysfunction, carotid arteriosclerosis, and other adverse events compared to metabolically healthy normal-weight individuals. These findings challenge the presumed benign nature of this phenotype [16,17,18].

Obesity is a heterogeneous condition that encompasses multiple phenotypes, including metabolically healthy obesity (MHO), a subtype characterized by variable definitions and a relatively favorable metabolic profile. Although it may appear “healthy”, numerous cohort studies have demonstrated that MHO is not free of risk, as it is associated with chronic diseases such as type 2 diabetes, hypertension, cardiovascular disease, chronic kidney disease, and certain types of cancer. Moreover, between 30% and 50% of individuals with MHO develop metabolic complications over time, highlighting the instability of the metabolically healthy phenotype. While some authors have suggested that MHO represents a group resistant to cardiometabolic deterioration, the majority of studies agree that this profile tends to worsen. Therefore, it should not be considered a benign condition. Future studies should categorize patients according to their obesity phenotype to optimize prevention and treatment strategies. These measures should be applied not only to individuals with obesity but also to those with normal weight who are at metabolic risk [19].

A major limitation in studying MHO lies in the absence of a universally accepted definition. Different studies have applied varying criteria to classify metabolic health, incorporating diverse markers such as fasting glucose, blood pressure, triglyceride and HDL-cholesterol levels, insulin sensitivity indices, and inflammatory biomarkers [20]. This definitional heterogeneity hinders cross-study comparisons and contributes to the wide variability in reported prevalence rates, which can range from 10% to over 30% among populations with obesity [21]. Additionally, cultural, ethnic, and age-related factors modulate the expression and distribution of the MHO phenotype, emphasizing the need for population-specific research and culturally tailored interventions [22].

From a pathophysiological perspective, several mechanisms have been proposed to explain the MHO phenotype. Among the most relevant are a higher proportion of subcutaneous fat compared to visceral fat, greater adipocyte expandability and functionality, a less pro-atherogenic inflammatory profile in adipose tissue, and preserved insulin sensitivity [23,24,25]. Recent genetic studies highlight that adipose tissue distribution—particularly fat accumulation in the gluteofemoral region—is a key factor in the MHO phenotype. These findings underscore that not only the amount of fat but also its location and function are critical. In individuals of Asian ethnicity, a tendency to accumulate visceral and ectopic fat is associated with reduced insulin sensitivity for a given BMI, increasing their metabolic risk. In addition, sociocultural factors such as diet and lifestyle contribute to the ethnic differences observed in the prevalence of MHO, suggesting the need for a personalized approach to metabolic risk assessment. Currently, the MHO concept is used as a valuable tool for cardiometabolic risk stratification and treatment personalization in clinical practice, contributing to more precise and effective care [26].

Genetic and epigenetic factors, gut microbiota composition, and levels of physical activity are also believed to influence metabolic responses to adiposity. MHO individuals often demonstrate higher cardiorespiratory fitness, increased physical activity, and healthier dietary habits compared to MUO counterparts, potentially conferring protection against metabolic impairments [11,27].

Despite these observations, MHO should not be regarded as a benign or risk-free condition. Numerous cohort studies have indicated that while MHO individuals exhibit a lower risk of T2DM and CVD relative to MUO individuals, their risk remains significantly elevated compared to metabolically healthy individuals of normal weight [28,29,30].

Recent publications emphasize that MHO is not free from health risks. Several studies have shown a higher incidence of chronic diseases in individuals with this phenotype compared to metabolically healthy normal-weight individuals. For instance, an increased risk of developing certain types of cancer has been reported in people with MHO [31], as well as a significantly higher prevalence of non-alcoholic fatty liver disease (NAFLD) compared to those with a non-obese, metabolically healthy phenotype [32]. Regarding chronic kidney disease (CKD), a recent meta-analysis by Iqbal et al. (2024) [33] demonstrated that obesity, regardless of metabolic status, is a significant risk factor for its development. In fact, MHO increases the risk of CKD by 40%. Furthermore, the phenotype of metabolically unhealthy normal-weight individuals (MUNW) is also associated with a high risk of CKD, despite a normal body weight, and warrants greater attention in clinical practice [33].

These findings underscore the importance of appropriate clinical monitoring and preventive interventions, even among those with ostensibly preserved metabolic function despite excess body weight.

Moreover, the binary classification of obesity into metabolically healthy and unhealthy subtypes may oversimplify the complex and dynamic nature of metabolic risk. Accordingly, more integrative and continuous approaches to assessing metabolic health have been proposed, incorporating both conventional and emerging biomarkers, as well as accounting for lifestyle and environmental modulators [34]. The use of advanced imaging techniques and comprehensive metabolic profiling—including assessments of visceral fat, ectopic fat deposition, and subclinical inflammation—may enhance risk stratification and guide the development of personalized treatment strategies [35,36].

Public health policies and clinical guidelines must also reconcile the MHO concept with the broader imperative to address the obesity epidemic. While not all individuals with obesity develop overt metabolic disease, the overall burden of obesity-related complications remains substantial. Preventive efforts aimed at promoting healthy eating, physical activity, weight maintenance, and early detection of metabolic dysfunction are critical, irrespective of BMI [37]. Furthermore, the heterogeneity of obesity reinforces the need for tailored interventions that align with individual metabolic profiles rather than relying solely on anthropometric cutoffs [38].

In summary, the phenomenon of metabolically healthy obesity represents an area of growing scientific interest with significant clinical and public health implications. Although the existence of a subset of individuals with preserved metabolic function despite obesity challenges conventional risk paradigms, accumulating evidence suggests that MHO does not confer long-term immunity to adverse outcomes [39]. A more sophisticated and dynamic understanding of obesity and metabolic health—grounded in rigorous definitions, longitudinal data, and mechanistic insights—is essential to inform effective prevention and treatment strategies. As research continues to unravel the intricate interplay between adiposity and metabolic function, abandoning simplistic categorizations in favor of individualized approaches to obesity management becomes increasingly imperative.

It is important to note that this study employed a cross-sectional design, which limits the ability to infer causality, as data were collected at a single time point, allowing only the identification of associations without establishing cause-and-effect relationships.

The objective of this study is to assess the level of Type 2 Diabetes Risk in a cohort of workers classified as MHO.

## 2. Methods

### 2.1. Study Design and Population

A cross-sectional descriptive analysis was conducted using data from routine occupational health examinations collected between January 2019 and June 2020. The sample included 68,884 obese Spanish workers (45,498 men and 23,386 women) employed across the primary, secondary, and tertiary economic sectors. A group of metabolically healthy non-obese individuals with a BMI < 30 kg/m^2^ (187,316 men and 130,724 women) was also included, serving as the reference group. Missing data were managed by excluding participants with incomplete records from the analysis. No imputation methods were applied.

### 2.2. Inclusion Criteria

Participants were eligible if they met the following criteria:

- Body mass index (BMI) equal to or greater than 30 kg/m^2^, indicating obesity;
- Aged between 18 and 69 years;
- Employed by one of the companies involved in the research;
- Provided informed consent to participate.

**Figure 1:**
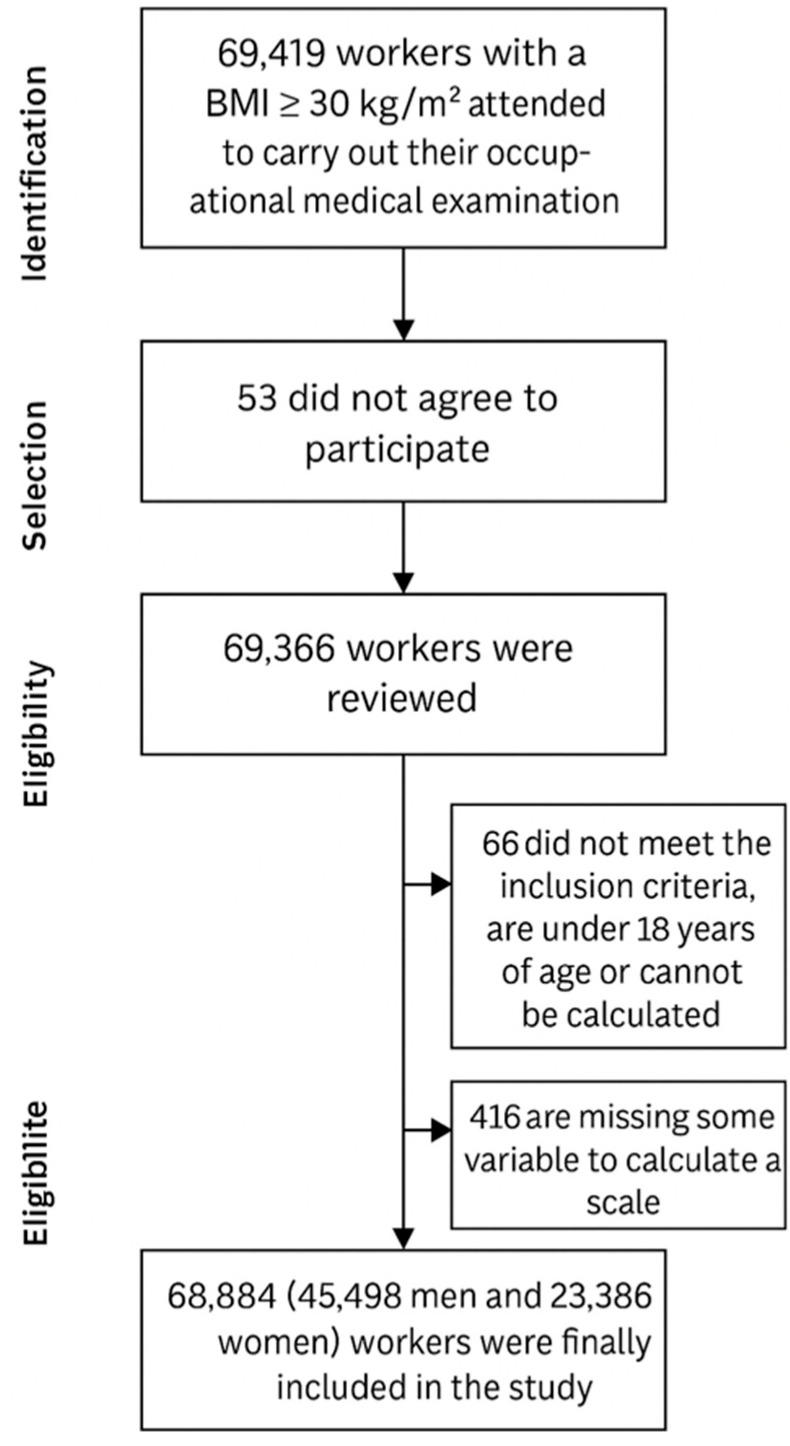
Flow diagram of participant inclusion.

A detailed flowchart depicting the participant selection process is provided in Figure 1.

### 2.3. Measurement of Variables

All clinical, biochemical, and anthropometric data were collected by trained medical and nursing staff, following prior standardization of measurement procedures. Height and weight were recorded using a SECA 700 stadiometer and scale (SECA, Chino, CA, USA). Waist circumference was measured with a SECA tape measure (SECA, Chino, CA, USA), with the subject standing upright, feet together, and abdomen relaxed. The tape was positioned horizontally at the level of the last floating rib.

Blood pressure was measured using a calibrated automatic sphygmomanometer (OMRON M3) (OMRON, Osaka, Japan) after the participant had rested in a seated position for at least 10 min. Three readings were taken at one-minute intervals, and the average value was used for analysis.

Following a minimum 12-h fast, venous blood samples were collected. Total cholesterol, fasting plasma glucose, and triglyceride levels were determined using automated enzymatic assays. High-density lipoprotein (HDL) cholesterol was measured using a dextran sulfate-MgCl_2_ precipitation method. Low-density lipoprotein (LDL) cholesterol was calculated indirectly via the Friedewald formula [40]:

LDL = Total cholesterol − HDL + (Triglycerides/5).

All lipid and glycemic parameters were reported in mg/dL.

Obesity was classified based on a BMI threshold of ≥30 kg/m^2^.

### 2.4. Definition of Metabolically Healthy Obesity (MHO)

MHO refers to individuals with obesity who, despite excess body fat, do not exhibit significant metabolic abnormalities such as insulin resistance, dyslipidemia, hypertension, or chronic inflammation. In contrast, metabolically unhealthy obesity (MUO) is characterized by the presence of these metabolic disturbances, which are associated with a higher risk of cardiovascular disease, type 2 diabetes, and other complications. This distinction enables more accurate risk stratification among individuals with obesity.

The classification of MHO was based on the metabolic syndrome (MetS) criteria established by the National Cholesterol Education Program Adult Treatment Panel III (NCEP ATP III) [41]. These include:

- Waist circumference ≥ 88 cm for women and ≥102 cm for men;
- Triglycerides ≥ 150 mg/dL or current treatment for elevated triglycerides;
- HDL-cholesterol < 50 mg/dL in women or <40 mg/dL in men;
- Fasting glucose ≥ 100 mg/dL or ongoing treatment for hyperglycemia;
- Systolic blood pressure ≥ 130 mmHg and/or diastolic ≥85 mmHg, or antihypertensive therapy.
- Participants were categorized into three MHO subgroups:
- Group A: No MetS components present;
- Group B: Presence of one MetS component;
- Group C: Presence of up to two MetS components.

The classification of MHO and its subgroups A, B, and C represents a progressive adaptation of the classical definition of metabolic syndrome. This segmentation enables a more precise identification of metabolic risk gradients among individuals with obesity, acknowledging that not all share the same risk profile. The subgroups reflect varying degrees of metabolic disturbances, allowing for improved stratification and understanding of the associated cardiometabolic risk.

Group C was included to reflect a progressively inclusive definition of MHO, acknowledging that individuals with up to two MetS components might still lack overt metabolic disease. This operational definition aligns with prior large cohort studies aiming to capture the MHO spectrum.

### 2.5. Sociodemographic and Lifestyle Variables

Sex was treated as a binary variable (male/female). Age was computed by subtracting the birthdate from the date of medical evaluation. Educational attainment was categorized into three levels: primary, secondary, and tertiary (university) education.

Socioeconomic status was defined according to the classification system established by the Spanish Society of Epidemiology, which is based on the 2011 National Classification of Occupations (CNO-11). Participants were grouped into three social classes: [42]

- Class I: Executives, university-trained professionals, athletes, and artists;
- Class II: Intermediate-level professionals and skilled self-employed workers;
- Class III: Low-skilled laborers.

Smoking status was assigned to any individual who had consumed any form of tobacco daily within the past 30 days or had quit smoking within the previous 12 months.

Adherence to the Mediterranean diet was evaluated through a 14-item questionnaire, awarding 0 or 1 point per item. A score ≥9 was indicative of high adherence [43].

Physical activity was assessed using the self-administered International Physical Activity Questionnaire (IPAQ), which quantifies activity during the previous seven days [44].

The assessment of type 2 diabetes risk in this study was performed using five validated predictive models [45], each incorporating a specific combination of clinical and demographic variables:

- Finrisk Score: This tool estimates diabetes risk based on several parameters, including age, gender, body mass index (BMI), waist circumference, levels of physical activity, consumption of fruits and vegetables, use of antihypertensive medication, previous episodes of hyperglycemia, and family history of diabetes. A score exceeding 15 is indicative of a high risk.
- QDiabetes Algorithm: This model incorporates variables such as age, sex, ethnicity, height, weight, fasting blood glucose, smoking status, prior stroke, family history of diabetes, use of blood pressure medications, mental health conditions (including schizophrenia or depression), and use of corticosteroids or statins. Additionally, a history of gestational diabetes or polycystic ovary syndrome is considered. Although this tool does not use a fixed threshold, in this study, a relative risk score of 3 or higher was defined as elevated.
- Canrisk Tool: This score is calculated based on factors such as age, sex, physical activity level, dietary intake of fruits and vegetables, personal history of hypertension or hyperglycemia, family history of diabetes, ethnic background, and educational level. Scores greater than 43 suggest a high probability of developing type 2 diabetes.
- TRAQ-D (Trinidad Risk Assessment Questionnaire for Diabetes): This tool requires input on age, gender, BMI, smoking habits, racial/ethnic background, and family history of diabetes to evaluate individual risk.
- Oman Diabetes Risk Score: This scale utilizes age, waist circumference, BMI, family history of diabetes, and current hypertension status to estimate risk.

### 2.6. Ethical Considerations

The study protocol adhered to the ethical principles outlined in the Declaration of Helsinki (2013 version). All data were anonymized and handled confidentially. The study received approval from the Research Ethics Committee of the Balearic Islands (reference number: IB 483/20).

Participant data were codified, ensuring that only the principal investigator could access identifying information. All researchers involved complied with the Spanish Organic Law 3/2018, of December 5, concerning the protection of personal data and digital rights. Participants retained the right to access, rectify, cancel, or oppose the use of their personal data at any time.

### 2.7. Statistical Analysis

Quantitative variables were analyzed using Student’s *t*-test, and results were reported as means with standard deviations. Categorical variables were analyzed using the chi-square test to estimate prevalence rates. A multinomial logistic regression model was employed to calculate odds ratios (ORs) with 95% confidence intervals (CIs). Statistical significance was set at *p* < 0.05. The Bonferroni correction was applied to address the issue of multiple comparisons and reduce the risk of type I errors. Analyses were conducted using IBM SPSS Statistics, version 28.0.

## 3. Results

Table 1 presents a comprehensive characterization of the study population stratified by obesity status and sex. Obese individuals, both men and women, exhibited significantly higher anthropometric, metabolic, and cardiovascular risk parameters compared to their non-obese counterparts (*p* < 0.001 for all variables). Notably, marked differences were observed in systolic and diastolic blood pressure, waist circumference, and triglyceride levels, underscoring the cardiometabolic burden associated with obesity. Additionally, adverse sociodemographic and lifestyle factors, including lower educational attainment, reduced physical activity, and limited adherence to a Mediterranean diet, were disproportionately prevalent among obese participants. These disparities highlight the complex interplay between social determinants and metabolic health, reinforcing the importance of context-specific preventive strategies.

Table 1 outlines the anthropometric and clinical characteristics of the study population. The group of individuals with obesity had a mean age of 42 years, compared to 39 years in the non-obese group. The obese group showed higher mean values for both blood pressure and lipid profile than the non-obese group. Individuals without obesity had a higher educational level, belonged to a more affluent social class, and reported healthier lifestyle habits.

Table 2 illustrates the mean values of six validated type 2 diabetes risk scores across MHO and MNHO phenotypes, stratified by sex and using three increasingly inclusive definitions of metabolic health. Across all scales and criteria, MNHO individuals exhibited significantly higher diabetes risk scores compared to MHO individuals (*p* < 0.001), irrespective of sex. The consistent gradient observed—with increasing risk from MHO to MNHO as definitions broaden—demonstrates the cumulative impact of metabolic syndrome components on diabetes risk estimation. Importantly, even MHO individuals displayed elevated scores compared to normative values, suggesting that obesity per se confers a residual metabolic risk despite preserved metabolic function. These findings underscore the importance of refining risk stratification tools to capture subtle gradations in metabolic impairment.

Table 3 reports the prevalence of high-risk classifications according to each diabetes risk scale across MHO and MNHO groups by sex. MNHO individuals consistently showed significantly higher proportions of high or very high diabetes risk compared to their MHO counterparts (*p* < 0.001), with this pattern holding across all definitions of metabolic health. The Finrisk and Canrisk scores demonstrated particularly marked differences, with the prevalence of high-risk individuals in MNHO women reaching nearly 48% and 43%, respectively, under the most inclusive definition (C). These data reinforce the enhanced predictive utility of integrating multiple metabolic risk factors, while also emphasizing that the MHO phenotype is not metabolically benign. Notably, a substantial subset of MHO individuals—especially men—still fell into moderate-to-high-risk categories, warranting targeted surveillance and preventive measures.

Table 4 presents the results of multinomial logistic regression analyses examining associations between sociodemographic, lifestyle, and metabolic variables and elevated diabetes risk across four validated scales. MNHO status was consistently and independently associated with significantly increased odds of high-risk classification across all models and definitions, with odds ratios ranging from 1.37 to 5.74. Other notable predictors included advancing age, male sex (particularly for Canrisk and TRAQ-D), low educational level, lower social class, absence of physical activity or Mediterranean diet, and smoking status. These findings not only validate the predictive capacity of traditional risk factors but also highlight the incremental risk conferred by MNHO status, independent of confounding variables. The robustness of these associations across multiple scales enhances the generalizability and clinical relevance of the findings.

## 4. Discussion

This large-scale cross-sectional study confirms that, although individuals classified as MHO exhibit a lower risk of type 2 diabetes mellitus (T2DM) than their MNHO counterparts, their risk remains substantially elevated compared to non-obese, metabolically healthy individuals. Across all six validated T2DM risk scores and under three increasingly inclusive definitions of metabolic health, MNHO individuals consistently presented with higher mean scores and a greater prevalence of high-risk categories. These findings support the accumulating evidence that MHO, while comparatively less harmful, does not represent a metabolically benign condition [46,47,48].

Importantly, a graded increase in diabetes risk was observed as definitions of MHO became more inclusive—moving from zero to two components of metabolic syndrome. This trend suggests a dose–response relationship between the accumulation of metabolic abnormalities and the estimated diabetes risk, consistent with previous findings that subclinical metabolic dysfunction confers risk even before the overt disease is established [49,50]. Notably, even MHO individuals with one or two MetS components demonstrated diabetes risk scores substantially above normative ranges, raising questions about the validity of the “healthy” label.

Sex-based disparities also emerged, with MNHO women showing particularly high-risk profiles in scales such as Finrisk and Canrisk. These differences may reflect sex-specific metabolic responses to adiposity, variations in fat distribution, hormonal milieu, or health behaviors [51,52]. Additionally, adverse sociodemographic factors—including low education, low occupational class, smoking, physical inactivity, and limited adherence to the Mediterranean diet—were independently associated with increased diabetes risk. These findings mirror previous research emphasizing the role of social determinants in shaping cardiometabolic health [53,54].

The strength of associations found in the multinomial logistic regression analyses—particularly the independent predictive value of the MNHO phenotype across all models—highlights the need to reassess the diagnostic and prognostic value of metabolic health classifications. Even after controlling for lifestyle, demographic, and socioeconomic confounders, MNHO status conferred between 1.37 and 5.74 times greater odds of high-risk classification. These results are consistent with longitudinal data showing elevated T2DM incidence in MHO individuals compared to metabolically healthy normal-weight controls [17,55].

From a clinical and public health perspective, several implications emerge. First, the notion that MHO individuals are protected from metabolic disease must be reconsidered, particularly in occupational health contexts where early prevention is critical. Second, this study reinforces calls to move beyond BMI-centric models of risk assessment toward multidimensional tools that integrate metabolic, behavioral, and social data [56,57]. Third, lifestyle interventions—especially those promoting physical activity and healthy diets—remain essential across all obesity phenotypes, given their strong association with diabetes risk.

These findings are especially relevant in the context of working-age adults, where routine occupational health screenings provide an ideal setting for early identification and intervention. Given the dynamic nature of the MHO phenotype, and its propensity to shift toward a metabolically unhealthy state over time [58], longitudinal monitoring and personalized risk assessment strategies are urgently needed.

The progressive aging of the global population is leading to increased public spending to meet the growing social and healthcare demands of older adults. One of the main determinants of healthcare costs is the overall health status of the population. To address this challenge, it is essential to develop effective public health interventions. Implementing policies focused on the prevention and management of modifiable risk factors—such as promoting health education across all socioeconomic levels and encouraging healthy lifestyle habits—is a key strategy. Initiating preventive actions early in life not only improves population health but also helps reduce healthcare expenditures and enhances overall quality of life. Moreover, these strategies have the potential to alleviate the long-term economic burden by preventing costly chronic diseases and improving healthcare system efficiency.

A key consideration is the role of public health policies in the prevention and management of the MHO phenotype. Government initiatives aimed at encouraging healthy lifestyles, improving access to nutritious foods, and promoting physical activity among workers may play a crucial role in reducing the progression of type 2 diabetes in this population. Collaboration among the health, education, and labor sectors could lead to more effective strategies for promoting long-term metabolic health.

To enhance employee health, several targeted actions can be implemented within the workplace. One key strategy is providing comprehensive health education training for all employees, which is essential for encouraging the adoption of healthy behaviors and preventing chronic diseases. Promoting healthy lifestyles should be supported by awareness programs tailored to the specific needs of each occupational group.

Additionally, implementing workplace nutrition policies is crucial for improving employees’ dietary habits. This includes offering healthy food options in company cafeterias and removing vending machines that promote the consumption of ultra-processed products. Ensuring work schedules that allow sufficient time for proper meals is also vital, helping to avoid rushed or nutritionally poor eating due to lack of breaks. Furthermore, providing on-site fitness facilities can encourage regular physical activity, leading to improvements in cardiovascular health and overall well-being.

Finally, organizations can implement programs focused on physical activity and stress management, such as mindfulness, yoga, and relaxation techniques. These interventions would not only enhance the health and well-being of employees but could also increase productivity and profitability by reducing absenteeism and improving employee performance.

### 4.1. Future Directions

Future studies should adopt longitudinal designs to better capture the progression from MHO to MNHO and to identify critical transition points. Including biomarkers such as HOMA-IR, TyG index, inflammatory cytokines, and imaging-based metrics of visceral and ectopic fat could significantly enhance risk stratification. Furthermore, consensus on operational definitions of MHO would improve cross-study comparisons and facilitate the development of evidence-based clinical guidelines [59,60].

### 4.2. Strengths and Limitations

#### 4.2.1. Strengths

This study benefits from an exceptionally large and well-characterized population-based sample, enhancing the statistical power and generalizability of the findings. The inclusion of multiple validated type 2 diabetes risk assessment tools (Finrisk, Canrisk, TRAQ-D, Thai, Oman, QD-score) allows for robust cross-validation of results and provides a multidimensional view of diabetes risk across different obesity phenotypes. The use of three definitions for metabolically healthy obesity adds methodological depth and sensitivity, capturing varying degrees of metabolic impairment. This approach, together with the use of validated instruments to assess physical activity levels and adherence to the Mediterranean diet, enhances the reliability of the study and offers a cost-effective and practical strategy for longitudinal evaluation and follow-up. Furthermore, the comprehensive adjustment for key sociodemographic, lifestyle, and dietary factors strengthens the internal validity of the associations observed.

#### 4.2.2. Limitations

Despite its strengths, the cross-sectional design of the study precludes causal inference and limits the ability to assess the long-term stability of the MHO phenotype or the progression to T2DM over time. The lack of direct biochemical markers such as insulin resistance indices (e.g., HOMA-IR, TyG, or METS-IR), inflammatory biomarkers, or imaging-based assessments of fat distribution may limit the metabolic profiling of participants. Additionally, the use of self-administered questionnaires may lead to recall bias or social desirability bias. To enhance validity, future studies are encouraged to incorporate objective tools such as pedometers and detailed dietary records. The absence of a universal definition for MHO complicates comparisons with other studies and may affect prevalence estimates.

Other potential confounding factors, such as comorbidities or pharmacological treatments, were not included due to the unavailability of these data. Additionally, the lack of biomarkers represents another limitation of our study, as they would have been valuable for enabling a more precise stratification of metabolic risk in this population.

## 5. Conclusions

In conclusion, while MHO individuals exhibit comparatively lower diabetes risk than MNHO individuals, they remain at a substantially elevated risk relative to non-obese, metabolically healthy individuals. These findings underscore the necessity of refining risk stratification approaches beyond BMI and adopting integrative models that incorporate metabolic, behavioral, and social determinants of health. Public health strategies and clinical guidelines should be reoriented to reflect the heterogeneity within obesity phenotypes, ensuring that all at-risk individuals—regardless of apparent metabolic health—receive appropriate monitoring and preventive care.

## Figures and Tables

**Table 1:** Characteristics of the population.

	Obese			Non-Obese		
	**Men n = 45,498**	**Women n = 23,386**		**Men n = 187,316**	**Women n = 130,724**	
	**Mean (SD)**	**Mean (SD)**	***p*-Value**	**Mean (SD)**	**Mean (SD)**	***p*-Value**
Age (years)	42.9 (10.0)	42.0 (10.4)	<0.001	39.0 (10.3)	38.8 (1.0)	<0.001
Height (cm)	173.2 (7.1)	160.0 (6.7)	<0.001	174.1 (7.0)	161.4 (6.5)	<0.001
Weight (kg)	99.7 (12.4)	87.5 (12.3)	<0.001	76.6 (9.9)	61.4 (8.7)	<0.001
BMI (kg/m2)	33.2 (3.2)	34.1 (3.9)	<0.001	25.3 (3.1)	23.6 (3.0)	<0.001
Waist (cm)	96.7 (8.9)	83.3 (8.8)	<0.001	85.5 (7.8)	72.2 (6.4)	<0.001
Hip (cm)	108.6 (7.9)	109.5 (9.3)	<0.001	98.0 (7.2)	95.1 (6.9)	<0.001
Systolic BP (mmHg)	131.8 (16.2)	124.0 (15.9)	<0.001	122.6 (14.2)	112.7 (13.9)	<0.001
Diastolic BP (mmHg)	81.0 (10.7)	76.9 (11.0)	<0.001	74.0 (10.2)	68.4 (9.6)	<0.001
Total cholesterol (mg/dL)	204.1 (38.8)	200.3 (37.4)	<0.001	193.9 (38.6)	192.4 (36.1)	<0.001
HDL-cholesterol (mg/dL)	48.3 (7.0)	51.2 (7.1)	<0.001	51.7 (6.9)	54.2 (7.6)	<0.001
LDL-cholesterol (mg/dL)	124.5 (37.5)	127.1 (37.0)	<0.001	119.5 (37.5)	121.4 (37.0)	<0.001
Triglycerides (mg/dL)	158.6 (108.4)	110.5 (55.8)	<0.001	115.3 (80.1)	84.1 (43.0)	<0.001
Glucose (mg/dL)	92.3 (14.0)	89.0 (13.4)	<0.001	87.1 (12.4)	83.3 (11.0)	<0.001
	**%**	**%**	*p*-value	%	%	*p*-value
<30 years	10.0	13.5	<0.001	19.8	20.5	<0.001
30–39 years	28.3	28.2		34.2	34.3	
40–49 years	34.5	32.3		28.5	28.9	
50–59 years	22.6	21.9		14.8	14.1	
60–69 years	4.6	4.3		2.7	2.2	
Elementary school	63.7	64.9	<0.001	60.6	49.4	<0.001
High school	32.3	30.6		34.5	42.5	
University	4.0	4.5		4.9	8.1	
Social class I	4.6	4.2	<0.001	5.4	7.5	<0.001
Social class II	15.7	21.4		17.9	35.3	
Social class III	79.7	74.4		76.7	57.2	
No physical activity	96.5	95.3	<0.001	43.4	38.5	<0.001
Yes physical activity	3.5	4.7		56.6	61.5	
No Mediterranean diet	91.8	85.1	<0.001	49.1	39.4	<0.001
Yes Mediterranean diet	8.2	14.9		50.9	60.6	
Non-smokers	68.3	74.0	<0.001	61.6	65.8	<0.001
Smokers	31.7	26.0		38.4	34.2	

Obese: Individuals with BMI > 30 kg/m^2^. Non-obese: metabolically healthy individuals with BMI <30 kg/m^2.^

**Table 2:** Mean values of diabetes type 2 risk scales according to MHO and MNHO by sex.

	n = 8764	n = 36,734		n = 24,264	n = 21,234		n = 34,660	n = 10,838	
	**MHO (A)**	**MNHO (A)**		**MHO (B)**	**MNHO (B)**		**MHO (C)**	**MNHO (C)**	
**Men**	**Mean (SD)**	**Mean (SD)**	***p*-Value**	**Mean (SD)**	**Mean (SD)**	***p*-Value**	**Mean (SD)**	**Mean (SD)**	***p*-Value**
Finrisk	8.6 (2.2)	11.1 (3.8)	<0.001	9.1 (2.6)	12.4 (3.9)	<0.001	9.9 (3.2)	13.2 (3.9)	<0.001
Canrisk	27.6 (5.8)	33.4 (8.8)	<0.001	29.3 (6.7)	35.6 (9.3)	<0.001	30.7 (7.7)	37.2 (9.3)	<0.001
Total	22.5 (10.8)	27.7 (12.4)	<0.001	24.6 (11.7)	29.0 (12.5)	<0.001	25.6 (12.1)	30.0 (12.4)	<0.001
TRAQ-D	7.1 (3.1)	8.8 (3.8)	<0.001	7.6 (3.4)	9.5 (3.8)	<0.001	7.9 (3.5)	10.2 (3.8)	<0.001
Thai	9.7 (1.9)	11.6 (2.3)	<0.001	10.6 (2.1)	11.9 (2.3)	<0.001	10.9 (2.2)	12.3 (2.3)	<0.001
Oman	7.9 (4.2)	11.1 (4.6)	<0.001	9.3 (4.4)	11.9 (4.7)	<0.001	9.9 (4.6)	12.5 (4.7)	<0.001
QD-score	2.9 (1.9)	3.5 (2.4)	<0.001	3.0 (2.0)	3.7 (2.6)	<0.001	3.2 (2.2)	4.0 (2.6)	<0.001
Women	n = 6146	n = 17,240		n = 14,446	n = 8938		n = 19,976	n = 3410	
Finrisk	9.0 (2.6)	11.1 (3.6)	<0.001	9.6 (2.9)	12.1 (3.7)	<0.001	10.0 (3.1)	13.5 (3.8)	<0.001
Canrisk	21.1 (5.7)	27.1 (8.6)	<0.001	23.1 (6.8)	29.6 (9.1)	<0.001	24.4 (7.5)	32.3 (9.9)	<0.001
Total	16.1 (9.2)	22.8 (12.5)	<0.001	18.6 (10.8)	25.0 (13.0)	<0.001	19.8 (11.4)	28.3 (13.3)	<0.001
TRAQ-D	5.1 (4.0)	7.1 (4.4)	<0.001	5.7 (4.2)	8.0 (4.4)	<0.001	6.2 (4.3)	8.8 (4.2)	<0.001
Thai	8.0 (3.2)	9.8 (3.6)	<0.001	8.7 (3.4)	10.3 (3.6)	<0.001	9.1 (3.5)	10.9 (3.7)	<0.001
Oman	8.1 (4.7)	11.2 (5.1)	<0.001	9.3 (5.0)	12.1 (5.0)	<0.001	10.0 (5.1)	12.8 (5.0)	<0.001
QD-score	4.1 (3.1)	4.6 (3.3)	<0.001	4.2 (3.0)	5.0 (3.5)	<0.001	4.4 (3.2)	5.2 (3.2)	<0.001

MHO Metabolically healthy obesity. MNHO Non-Metabolically healthy obesity. Notably, (A) 0 factors of metabolic syndrome. (B) < 2 factors of metabolic syndrome (C) < 3 factors of metabolic syndrome. TRAQ-D Trinidad Risk Assessment Questionnaire for Diabetes.

**Table 3:** Prevalence of high values of diabetes type 2 risk scales according to MHO and MNHO by sex.

	n = 8764	n = 36,734		n = 24,264	n = 21,234		n = 34,660	n = 10,838	
	**MHO (A)**	**MNHO (A)**		**MHO (B)**	**MNHO (B)**		**MHO (C)**	**MNHO (C)**	
**Men**	**%**	**%**	***p*-Value**	**%**	**%**	***p*-Value**	**%**	**%**	***p*-Value**
Finrisk high-very high	1.3	15.9	<0.001	2.5	24.5	<0.001	7.2	30.9	<0.001
Canrisk high	18.0	47.8	<0.001	28.3	57.7	<0.001	35.0	64.7	<0.001
TRAQ-D high-very high	7.3	12.9	<0.001	6.8	16.5	<0.001	8.5	20.2	<0.001
QD-score ≥ 3	30.1	45.1	<0.001	35.1	50.2	<0.001	37.7	56.7	<0.001
Women	n = 6146	n = 17,240		n = 14,446	n = 8938		n = 19,976	n = 3410	
Finrisk high-very high	1.8	17.7	<0.001	4.9	27.5	<0.001	8.6	47.6	<0.001
Canrisk high	4.9	24.0	<0.001	10.5	32.7	<0.001	14.9	42.8	<0.001
TRAQ-D high-very high	4.0	9.4	<0.001	5.4	12.2	<0.001	6.9	14.1	<0.001
QD-score ≥ 3	58.1	67.0	<0.001	60.0	72.1	<0.001	62.7	76.2	<0.001

MHO Metabolically healthy obese. MNHO Metabolically non-healthy obese. (A) 0 factors of metabolic syndrome. (B) < 2 factors of metabolic syndrome (C) < 3 factors of metabolic syndrome. TRAQ-D Trinidad Risk Assessment Questionnaire for Diabetes.

**Table 4:** Multinomial logistic regression.

	Finrisk High-Very High	Canrisk High	TRAQ-D High-Very High	QD-Score ≥ 3
	**OR (95% CI)**	**OR (95% CI)**	**OR (95% CI)**	**OR (95% CI)**
Female	1	1	1	1
Male	0.69 (0.66–0.73)	5.25 (4.98–5.53)	1.20 (1.13–1.28)	0.64 (0.58–0.70)
<30 years	1	1	1	1
30–39 years	1.57 (1.43–1.73)	1.28 (1.21–1.35)	1.18 (1.11–1.25)	1.35 (1.23–1.47)
40–49 years	3.57 (3.25–3.92)	1.88 (1.60–2.16)	1.72 (1.42–2.03)	1.49 (1.30–1.69)
50–59 years	8.16 (7.33–9.09)	2.22 (1.90–2.52)	2.10 (1.71–2.50)	1.90 (1.61–2.20)
60–69 years	11.65 (9.89–13.71)	4.30 (3.78–4.88)	2.94 (2.68–3.22)	2.33 (1.90–2.77)
University	1	1	1	1
High school	1.10 (1.05–1.15)	1.54 (1.45–1.64)	1.24 (1.14–1.35)	1.18 (1.12–1.25)
Elementary school	2.26 (2.05–2.52)	3.28 (2.50–4.06)	2.02 (1.67–3.37)	1.60 (1.42–1.79)
Social class I	1	1	1	1
Social class II	1.05 (1.02–1.08)	1.19 (1.11–1.27)	1.30 (1.18–1.42)	1.23 (1.15–1.31)
Social class III	2.57 (2.17–2.97)	2.40 (2.01–2.80)	2.21 (1.81–2.62)	1.78 (1.58–1.99)
Yes physical activity	1	1	1	1
No physical activity	6.88 (6.12–7.64)	5.15 (4.60–5.71)	2.89 (2.50–3.29)	4.23 (3.60–4.87)
Yes Mediterranean diet	1	1	1	1
No Mediterranean diet	4.29 (3.90–4.69)	2.79 (2.50–3.09)	1.90 (1.48–2.33)	2.40 (2.01–2.80)
Smokers	1	1	1	1
Non-smokers	1.13 (1.08–1.18)	2.18 (2.04–2.34)	5.31 (5.01–5.63)	1.55 (1.38–1.72)
MHO (A)	1	1	1	1
MNHO (A)	5.74 (4.72–6.76)	2.17 (2.05–2.29)	1.41 (1.28–1.55)	1.42 (1.36–1.49)
MHO (B)	1	1	1	1
MNHO (B)	3.96 (3.68–4.24)	2.50 (2.36–2.66)	1.59 (1.48–1.71)	1.37 (1.31–1.43)
MHO (C)	1	1	1	1
MNHO (C)	2.63 (2.48–2.79)	2.85 (2.40–3.30)	1.42–1.33–1.52)	1.57 (1.49–1.65)

MHO Metabolically healthy obese. MNHO Metabolically non-healthy obese. (A) 0 factors of metabolic syndrome. (B) < 2 factors of metabolic syndrome (C) < 3 factors of metabolic syndrome. TRAQ-D Trinidad Risk Assessment Questionnaire for Diabetes. OR Odds ratio.

## Data Availability

Due to ethical and privacy considerations, access to the dataset is restricted. The data are stored in a secure database managed by ADEMA University School. The institution’s Data Protection Officer is Ángel Arturo López González.
